# Deciphering early events involved in hyperosmotic stress-induced programmed cell death in tobacco BY-2 cells

**DOI:** 10.1093/jxb/ert460

**Published:** 2014-01-13

**Authors:** Emanuela Monetti, Takashi Kadono, Daniel Tran, Elisa Azzarello, Delphine Arbelet-Bonnin, Bernadette Biligui, Joël Briand, Tomonori Kawano, Stefano Mancuso, François Bouteau

**Affiliations:** ^1^Université Paris Diderot, Sorbonne Paris Cité, Institut des Energies de Demain (UMR8236), Paris, France; ^2^Institut de Biologie des Plantes, Bât 630, 91405 Orsay, France; ^3^LINV-DiSPAA, Department of Agri-Food and Environmental Science, University of Florence, Viale delle Idee 30, 50019 Sesto Fiorentino (FI), Italy; ^4^Graduate School of Environmental Engineering, University of Kitakyushu 1-1, Hibikino, Wakamatsu-ku, Kitakyushu 808-0135, Japan; ^5^Laboratory of Crop Science, Department of Plant Resources, Faculty of Agriculture, Kyushu University, 6-10-1 Hakozaki, Higashi-ku, Fukuoka 812–8581, Japan; ^6^University of Florence LINV Kitakyushu Research Center (LINV@Kitakyushu), Kitakyushu, Japan; ^7^Université Paris Diderot, Sorbonne Paris Cité, Paris Interdisciplinary Energy Research Institute (PIERI), Paris, France

**Keywords:** Calcium, hyperosmotic stress, mitochondria, NaCl, Nicotiana tabacum, non-selective cation channels, programmed cell death, reactive oxygen species.

## Abstract

Hyperosmotic stresses represent one of the major constraints that adversely affect plants growth, development, and productivity. In this study, the focus was on early responses to hyperosmotic stress- (NaCl and sorbitol) induced reactive oxygen species (ROS) generation, cytosolic Ca^2+^ concentration ([Ca^2+^]_cyt_) increase, ion fluxes, and mitochondrial potential variations, and on their links in pathways leading to programmed cell death (PCD). By using BY-2 tobacco cells, it was shown that both NaCl- and sorbitol-induced PCD seemed to be dependent on superoxide anion (O_2_·^–^) generation by NADPH-oxidase. In the case of NaCl, an early influx of sodium through non-selective cation channels participates in the development of PCD through mitochondrial dysfunction and NADPH-oxidase-dependent O_2_·^–^ generation. This supports the hypothesis of different pathways in NaCl- and sorbitol-induced cell death. Surprisingly, other shared early responses, such as [Ca^2+^]_cyt_ increase and singlet oxygen production, do not seem to be involved in PCD.

## Introduction

Salt stress is known to have severe effects on plant growth and development (Tester and Davenport, 2003). High salinity leads to ionic, osmotic, and oxidative stress in plants (Zhu, 2001) that may result in the induction of signalling events that lead to programmed cell death (PCD) in higher plants (Huh *et al.*, 2002; Lin *et al.*, 2006; Shabala, 2009; Wang *et al.*, 2010) and algae (Affenzeller *et al.*, 2009). Such PCD could be regarded as a salt adaptation mechanism (Huh *et al.*, 2002). Drought, which consists at least in part of a hyperosmotic stress, was also shown to induce PCD in plants (Duan *et al.*, 2010). PCD is an active cellular process that facilitates the removal of unwanted or damaged cells and is essential for cellular differentiation and tissue homeostasis (van Doorn *et al.*, 2011). PCD has effectively been proved to occur in response to various abiotic stresses (Kadono *et al.*, 2010; van Doorn *et al.*, 2011). Different types of PCD with overlapping morphological and physiological hallmarks have been described in plants, which has led to a call for a detailed classification of cell death events (van Doorn, 2011; van Doorn *et al.*, 2011). Although the delineation between the different PCD types of sometimes remains difficult (van Doorn *et al.*, 2011), typical hallmarks of PCD in plants frequently include the fragmentation of the DNA by specific nucleases (DNA laddering), condensation and shrinkage of the cytoplasm, release of cytochrome *c* from mitochondria, elevation of the cytosolic calcium concentration ([Ca^2+^]_cyt_), generation of reactive oxygen species (ROS), and an activity increase of caspase-like enzymes (van Doorn, 2011; Tsiatsiani *et al.*, 2011).

Although early events reported in responses to ionic and non-ionic hyperosmotic stress such as a transient [Ca^2+^]_cyt_ increase (Xiong *et al.*, 2002; Donaldson *et al.*, 2004; Lin *et al.*, 2006; Kim *et al.*, 2007; Parre *et al.*, 2007; Ranf *et al.*, 2008), generation of ROS (Zhu, 2001; Xiong *et al.*, 2002; Lin *et al.*, 2006; Zhang *et al.* 2013), or up-regulation of protein kinases (Zhang *et al.*, 2013) seemed to be shared in plants, some other responses are specific to one of these stresses. Under saline conditions, Na^+^ enters the cells through non-selective cation channels (NSCCs; Demidchik and Tester, 2002), depolarizing the plasma membrane (Chen *et al.*, 2007; Hua *et al.*, 2008; Shabala and Cuin, 2008; Pandolfi *et al.*, 2010; Wegner *et al.*, 2011). In contrast, isotonic mannitol or sorbitol solutions cause significant membrane hyperpolarization (Li and Delrot, 1987; Zingarelli *et al.*, 1999; Shabala *et al.*, 2000; Shabala and Lew, 2002). The DNA laddering, due to endonuclease release through permeable transition pores (PTPs) leading to mitochondria depolarization (Huh *et al.*, 2002; Lin *et al.*, 2006) occurred in NaCl- but not in sorbitol-stressed cells (Affenzeller *et al.*, 2009). Thus, although some common events are induced upon osmotic stress, multiple signal transduction pathways are involved in the response to ionic and non-ionic hyperosmotic treatments (Donaldson *et al.*, 2004; Parré *et al*., 2007).

In this study, it was shown using Bright Yellow 2 (BY-2) cells that both ionic and non-ionic hyperosmotic stresses effectively induced early singlet oxygen (^1^O_2_) generation and an ^1^O_2_-dependent influx of Ca^2+^, which are not involved in PCD processes. The PCD observed in response to NaCl and sorbitol seemed to be dependent on delayed superoxide anion (O_2_·^–^) generation by NADPH-oxidase, this last being linked to Na^+^ influx through NSCCs and mitochondrial dysfunction only in the case of NaCl hyperosmotic stress.

## Materials and methods

### Cell culture conditions

*Nicotiana tabacum* L. BY-2 suspension cells were grown in Murashige and Skoog (MS) medium, pH 5.8 augmented with 30g l^–1^ sucrose and 0.2mg l^–1^ 2,4 D (Pauly *et al.*, 2001). Cells were maintained at 22±2 °C, under continuous darkness and continuous shaking (gyratory shaker) at 120rpm. Cell suspensions were subcultured weekly using a 1:15 dilution. All experiments were performed at 22±2 °C using log-phase cells (6 d after subculture) maintained in their culture medium. Cell density was ~4×10^5^ cells ml^–1^.

### Osmolality changes

The osmolality changes were systematically obtained by addition of 50 μl of sorbitol or NaCl from various stock solutions. For the measurement of extracellular medium osmolality changes after NaCl or sorbitol treatment, 100 μl of supernatant of cell suspensions treated with NaCl or sorbitol, were determined by the freezing depression method using an Automatic Micro-Osmometer Type 15 (Löser Messtechnik, Berlin, Germany).

### Cell viability assays

Hyperosmosis-induced cell death in the cell suspension culture was determined by staining the dead cells with the vital dye Evans blue (0.005%, w/v) by mixing and incubating the cells and the dye for 10min. Then stained cells were observed under a microscope. When appropriate, a 15min pre-treatment with pharmacological effectors was done prior to NaCl or sorbitol exposure. Cells were counted under a microscope and cells that accumulated Evans blue were considered dead. At least 500 cells were counted for each independent treatment, and the procedure was repeated at least three times for each condition.

### Monitoring of ROS production

The production of ROS was monitored by the chemiluminescence of the *Cypridina* luciferin analogue (CLA) as previously described (Kadono *et al.*, 2006, 2010). CLA is known to react mainly with O_2_·^–^ and^ 1^O_2_ with light emission (Nakano, 1986), and allows measurement of extracellular ROS in plant cells (Tran *et al.*, 2013). Chemiluminescence from CLA was monitored using an FB12-Berthold luminometer (with a signal integrating time of 0.2 s). For data analysis, the luminescence ratio (L/L_basal_) was calculated by dividing the intensity of CLA luminescence (L) by the luminescence intensity before treatment (L_basal_). The ROS scavengers 1,2-dihydroxybenzene-3,5-disulphonic acid disodium salt (Tiron), 1,4-diazabicyclo[2.2.2]octane (DABCO), and salicylhydroxamic acid (SHAM) were added 5min before NaCl and sorbitol treatment. Other inhibitors were added to the cell suspension 30min before NaCl and sorbitol treatment.

### Aequorin luminescence measurements

The [Ca^2+^]_cyt_ variations were recorded in a BY-2 cell suspension expressing the aequorin gene. Aequorin was reconstituted by overnight incubation in MS medium supplemented with 30g l^–1^ sucrose and 2.5 μM native coelenterazine. Cell culture aliquots (500 μl) were transferred carefully into a luminometer tube, and the luminescence counts were recorded continuously at 0.2 s intervals with a luminometer. Treatments were performed by pipette injection of 50 μl of the effectors (NaCl or sorbitol). The residual aequorin was discharged by addition of 500 μl of a 1M CaCl_2_ solution dissolved in 100% methanol. The resulting luminescence was used to estimate the total amount of aequorin in each experiment. Calibration of calcium measurement was performed by using the equation: pCa=0.332588(–log*k*)+5.5593, where *k* is a rate constant equal to luminescence counts per second divided by the total remaining counts (Knight *et al.*, 1996). The results are expressed in micromolar Ca^2+^ and correspond to the mean±SD of 3–5 independent experiments.

### Voltage clamp measurements

Experiments were conducted on 6-day-old cells maintained in their culture medium to limit stress (main ions in MS medium 28mM NO_3_^–^, 16mM K^+^). Individual cells were immobilized by a microfunnel (~50–80 μm outer diameter) and controlled by a micromanipulator (WR6-1, Narishige, Japan). Impalements were carried out with a piezoelectric micromanipulator (PCS-5000, Burleigh Inst., USA) in a chamber (500 μl) made of Perspex. Voltage clamp measurements of whole-cell currents from intact cultured cells presenting a stable running membrane potential were carried out using the technique of the discontinuous single voltage clamp microelectrode (dSEVC; Finkel and Redman, 1984). In this technique, both current passing and voltage recording use the same microelectrode. Interactions between the two tasks are prevented by time-sharing techniques (sampling frequency 1.5–3kHz). Microelectrodes were made from borosilicate capillary glass (Clark GC 150F, Clark Electromedical, Pangbourne Reading, UK) pulled on a vertical puller (Narishige PEII, Japan). Their tips were <1 μm diameter; they were filled with 600mM KCl, and had electrical resistances between 20 MΩ and 50 MΩ with the culture medium. The capacity compensation of the microelectrode amplifier (Axoclamp 2A, Molecular Devices, Sunnyvale, CA, USA) was set to a subcritical level to produce the fastest electrode response. The relatively large size of the cells ensured a sufficiently high membrane time constant despite a relatively low input resistance (~40 MΩ). Specific software (pCLAMP 8) drives the voltage clamp amplifier. Voltage and current were simultaneously displayed on a dual input oscilloscope (Gould 1425, Gould Instruments Ltd, Hainault, UK), digitalized with a Digidata 1322A (Molecular Devices). In whole-cell current measurements, the membrane potential was held to the value of the resting membrane potential. Current recordings were obtained by hyperpolarizing pulses from –200 mV to +80 mV (20 mV, 2 s steps of current injection, 6 s of settling time). It was systematically checked that cells were correctly clamped by comparing the protocol voltage values with those really imposed. Only microelectrodes presenting a linear relationship were used.

### Confocal microscopy

Confocal imaging was performed using an upright Leica Laser Scanning Confocal Microscope SP5 (Leica Microsystems, Germany) equipped with a ×63 oil immersion objective. To analyse the NaCl influx, the sodium indicator Sodium Green was used (Molecular Probes, USA). The BY-2 tobacco cells were pre-incubated for 15min with an NSCC inhibitor and then incubated with 200mM NaCl for 1h. Sodium Green indicator (10 μM) was added to the solution 30min after the beginning of the salt treatment. After incubation with NaCl and Sodium Green indicator, the BY-2 cells were washed with phosphate-buffered saline (PBS) buffer. The excitation wavelength was set at 514nm, and the emission was detected at 530±20nm.

### Mitochondrial membrane potential measurement

Six-day-old BY-2 suspension cells were collected and washed by filtration in a suspension buffer containing 50mM HEPES, 0.5mM CaCl_2_, 0.5mM K_2_SO_4_, and 10mM glucose, pH 7.0 (Errakhi *et al.*, 2008). After treatment, cells were stained with the mitochondrial membrane potential probe JC-1 by incubating 2ml of cell suspensions for 15min (24 °C in the dark) with 2 μg ml^–1^ JC-1 (3 μM). JC-1 was dissolved and stored according to the manufacturer’s instructions. Treated cells without prior washing were subjected to analysis using a Hitachi F-2000 fluorescence spectrophotometer. The excitation wavelength used was 500nm. Fluorescence signals were collected using a band pass filter centred at 530nm and 590nm.

### Chemicals

All chemical products were purchased from Sigma-Aldrich (Saint-Quentin Fallavier, France), except JC1 and Sodium Green indicator which were from Molecular Probes (Saint Aubin, France). Stock solution of diphenyleneiodonium chloride (DPI; 10mM) was dissolved in dimethylsulphoxide (DMSO) in order to obtain a 0.01% final concentration of DMSO. This DMSO concentration did not induce any change in ROS or [Ca^2+^]_cyt_ levels (not shown). All other chemicals were dissolved in water.

### Statistical analysis

Data were analysed by analysis of variance (ANOVA), and the mean separation was achieved by Newman and Keuls multiple range test. All numerical differences in the data were considered significantly different at the probability level of *P* ≤ 0.05.

## Results

### Hyperosmotic changes induce cell death in *N. tabacum* BY-2 suspension-cultured cells

**Fig. 1.**
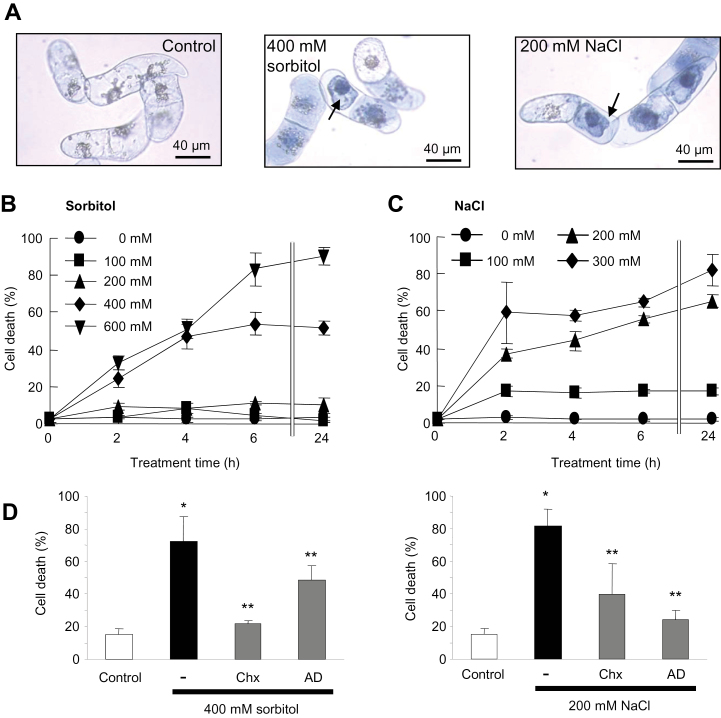
NaCl- and sorbitol-induced cell death in tobacco BY-2 cells. (A) Light micrographs of BY-2 cultured cells stained with Evans blue 2h after incubation with 400mM sorbitol (centre) or 200mM NaCl (right) compared with control cells maintained in their medium (left). Arrows indicate the cell shrinkage. (B) Effect of incubation time and concentration of sorbitol or NaCl on the extent of cell death. (C) Effect of pre-treatment with actinomycin D (AD; 20 μg ml^–1^) or cycloheximide (Chx; 20 μg ml^–1^) on sorbitol- and NaCl-induced cell death. Each data point and error bar reflect the mean and SD, respectively, of at least three independent replicates. *Significantly different from controls, *P* < 0.05; **significantly different from the NaC-l or sorbitol-treated cells, *P* < 0.05. (This figure is available in colour at *JXB* online.)

The impact of NaCl and sorbitol additions on osmolality changes in BY-2 medium was first evaluated and it was found that the concentrations of NaCl (200mM) and sorbitol (400mM) most frequently used in this study showed almost the same osmolality shifts (Table 1). These shifts in osmolality induced by 400mM sorbitol or 200mM NaCl led to the death of a part of the cell population, dead cells displaying large cell shrinkage (Fig. 1A), the hallmark of the PCD process (van Doorn, 2011). Cell death scoring at various concentrations of sorbitol and NaCl showed the time- and dose-dependent progression of death (Fig. 1B, C), half of the cells being dead after 4h at 400mM sorbitol and 200mM NaCl. In order to confirm whether this cell death was due to an active process requiring active gene expression and cellular metabolism, BY-2 cell suspensions were treated with actinomycin D (AD), an inhibitor of RNA synthesis, or with cycloheximide (Chx), an inhibitor of protein synthesis, each at 20mg ml^–1^, 15min prior to 200mM NaCl or 400mM sorbitol exposure. In both cases, AD and Chx significantly reduced cell death (Fig. 1D). These results indicated that this cell death required active cell metabolism, namely gene transcription and *de novo* protein synthesis. Taken together, these data showed that saline or non-saline hyperosmotic stress induced a rapid PCD of a part of the *N. tabacum* BY-2 suspension cell population.

**Table 1.** Osmolality changes in the medium after treatment with NaCl and sorbitol

	Medium	NaCl (mM)			Sorbitol (mM)		
		100	200	300	200	400	600
Osmolality (mosmol)	182	369	558	944	375	605	952

The kinetics of some early events classically detected upon saline stress or drought, namely an increase in cytosolic Ca^2+^, ion flux variations, ROS production, and mitochondrial membrane depolarization, were then followed, and it was checked how they could be involved in PCD induced by hyperosmotic stress.

### Sorbitol- and NaCl-induced ROS generation

**Fig. 2.**
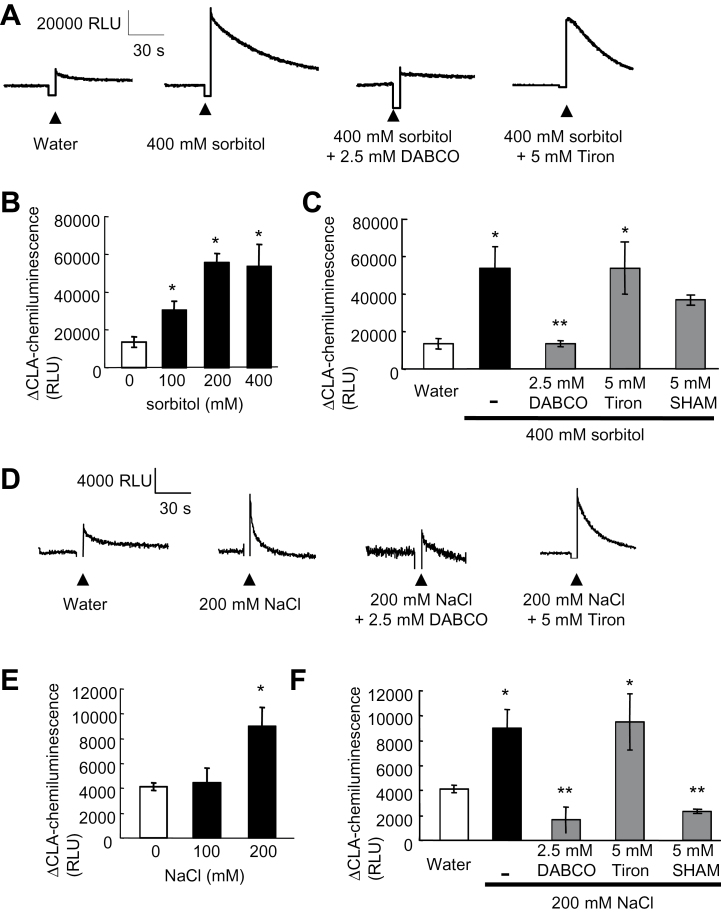
Induction of rapid ROS generation in tobacco BY-2 cells by sorbitol or NaCl. (A) Typical kinetics of the sorbitol-induced increase in CLA chemiluminescence reflecting the production ROS and modulation by ROS scavengers. (B) Effect of the concentration of sorbitol on ROS generation. (C) Modulation of sorbitol-induced ROS generation by DABCO, a scavenger of singlet oxygen, Tiron, a scavenger of the superoxide anion, or salicylhydroxamic acid (SHAM), an inhibitor of peroxidase. (D) Typical kinetics of the NaCl-induced increase in CLA chemiluminescence and modulation by ROS scavengers. (E) Effect of the concentration of NaCl on ROS generation. (F) Modulation of NaCl-induced ROS generation by DABCO, Tiron, or SHAM. Each data point and error bar reflect the mean and SD, respectively (*n*=5). *Significantly different from controls, *P* < 0.05; **significantly different from the NaCl- or sorbitol-treated cells, *P* < 0.05.

To study the effect of sorbitol on production of ROS in BY-2 cell suspension culture, the chemiluminescence of CLA, which indicates the generation of O_2_·^–^ and ^1^O_2_, was used. Addition of 400mM sorbitol to BY-2 cell suspension culture resulted in transient production of ROS that reaches the maximal level immediately after treatment (Fig. 2A). This sorbitol-induced ROS generation was dose dependent (Fig. 2B) and could be blocked using DABCO, an ^1^O_2_ scavenger, but not Tiron, an O_2_·^–^ scavenger (Fig. 2A, C). Addition of 200mM NaCl to BY-2 cell suspension culture also resulted in transient production of ROS that reaches the maximal level immediately after NaCl treatment (Fig. 2D, E). In the case of sorbitol, only DABCO was able to decrease the NaCl-induced CLA chemiluminescence (Fig. 2D, F). Thus, in both cases the early increase in CLA chemiluminescence seemed to be dependent on ^1^O_2_ generation but not on O_2_·^–^ generation. SHAM, an inhibitor of peroxidase (POX) (Kawano *et al.*, 1998; Hossain *et al.*, 2013), which could be responsible for extracellular ^1^O_2_ generation (Kawano *et al.*, 1998; Kanofsky, 2000; Guo *et al.*, 2009), was thus used. Pre-treatment of the BY-2 cell suspension culture with 5mM SHAM only slightly reduced the increase in CLA chemiluminescence induced by 400mM sorbitol (Fig. 2C) but significantly reduced that induced by 200mM NaCl (Fig. 2F). This suggests the involvement of POX, at least in NaCl-induced ^1^O_2_ generation.

**Fig. 3.**
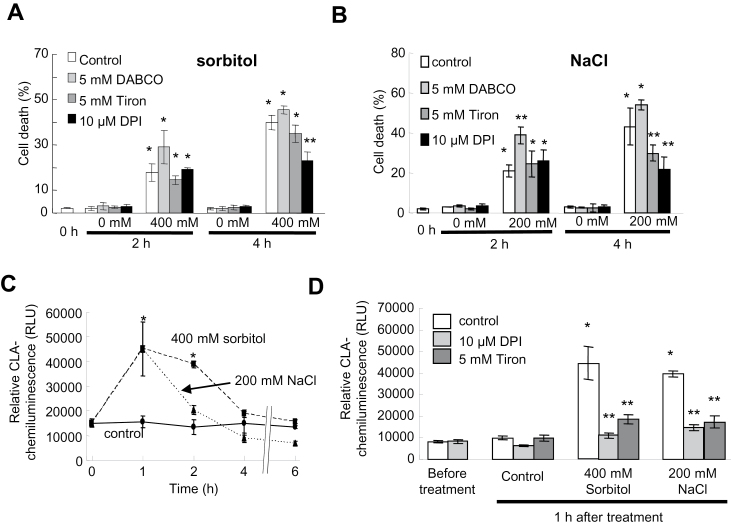
Effects of ROS scavengers on NaCl- or sorbitol-induced cell death in BY-2 cells. (A) Effect of Tiron, DABCO, or DPI, an NADPH-oxidase inhibitor, on cell death induced by 400mM sorbitol after 2h or 4h treatment. (B) Effect of Tiron, DABCO, or DPI on cell death induced by 200mM NaCl after 2h or 4h treatment. (C) Time course of CLA chemiluminescence during 6h treatment with 400mM sorbitol or 200mM NaCl. (D) Inhibition of sorbitol and NaCl-induced delayed ROS generation by Tiron or DPI. Each data point and error bar reflect the mean and SD, respectively (*n*=3). *Significantly different from controls, *P* < 0.05; **significantly different from the NaCl- or sorbitol-treated cells, *P* < 0.05.

The impact of ROS pharmacology on NaCl- and sorbitol-induced PCD (Fig. 1) was further checked. DABCO, the ^1^O_2_ scavenger, failed to decrease sorbitol- (400mM) and NaCl- (200mM) induced cell death and even increased NaCl-induced cell death after 2h of treatment (Fig. 3A, B). For Tiron, the O_2_·^–^ scavenger, there was no effect after 2h but a decrease in cell death could be observed after 4h treatment with NaCl (Fig. 3B). Thus, the hyperosmotic stress-induced cell death seemed not to be dependent on ^1^O_2_ generation but on O_2_·^–^ generation. Thus a possible delayed O_2_·^–^ generation after treatment with 400mM sorbitol or 200mM NaCl was searched for. After such hyperosmotic stress, increases in CLA chemiluminescence could be detected (Fig. 3C). In both cases of CLA chemiluminescence increases, the maximum chemiluminescence levels were reached after 1h and then decreased to the control level after 4h, the decrease being more rapid upon NaCl treatement (Fig. 3C). It is noteworthy that these increases occurred before cell death reached the plateau level (Fig. 1B, C). These increases in CLA chemiluminescence could be inhibited by pre-treatment with Tiron, but also with 10 μM DPI, an inhibitor of NADPH-oxidase (Fig. 3D), suggesting that the generation O_2_·^–^ through enhancement of NADPH-oxidase activity was involved in the delayed ROS generation after treatment with sorbitol and NaCl (Fig. 3C). In agreement with this, 10 μM DPI could also significantly reduce both sorbitol- and NaCl-induced cell death after 4h (Fig. 3A, B).

### Sorbitol and NaCl induce a rapid change in [Ca^2+^]_cyt_

**Fig. 4.**
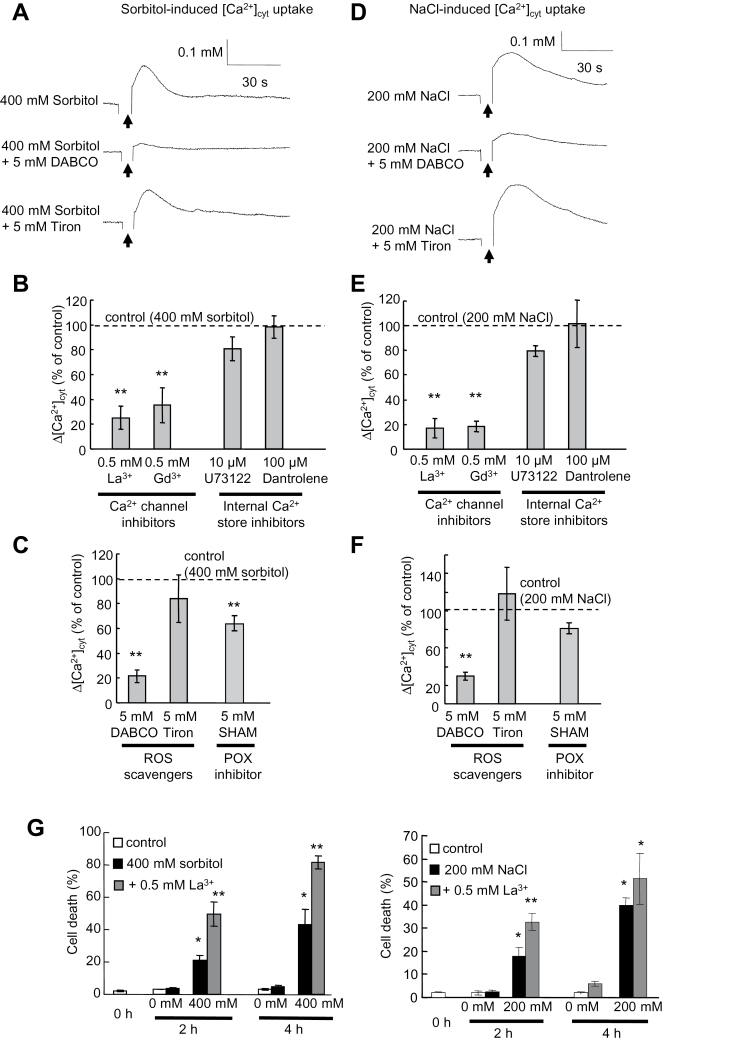
Induction of [Ca^2+^]_cyt_ increase in aequorin-expressing tobacco BY-2 cells by sorbitol or NaCl. (A) Typical kinetics of sorbitol-induced increase in [Ca^2+^]_cyt_ and modulation by ROS scavengers. (B) Modulation of sorbitol-induced [Ca^2+^]_cyt_ increase by the calcium channel blockers La^3+^ or Gd^3+^ (500 μM each) and the internal stock release inhibitors U73122 (100 μM) and dantrolene (10 μM). (C) Modulation of sorbitol-induced [Ca^2+^]_cyt_ increase by DABCO, a scavenger of singlet oxygen, Tiron, a scavenger of anion superoxide, or salicylhydroxamic acid (SHAM), an inhibitor of peroxidase. (D) Typical kinetics of the NaCl-induced increase in [Ca^2+^]_cyt_ and modulation by ROS scavengers. (E) Modulation of the NaCl-induced [Ca^2+^]_cyt_ increase by La^3+^, Gd^3+^ (500 μM each), U73122 (100 μM), or dantrolene (10 μM). (F) Modulation of NaCl-induced [Ca^2+^]_cyt_ increase by DABCO, Tiron, or SHAM. (G) Effect of La^3+^ on cell death induced by 200mM NaCl (left) or 400mM sorbitol (right) after 2h or 4h treatment. Each data point and error bar reflect the mean and SD, respectively (*n*=5). *Significantly different from controls, *P* < 0.05; **significantly different from the NaCl- or sorbitol-treated cells, *P* < 0.05.

The changes in [Ca^2+^]_cyt_ were monitored by the Ca^2+^-dependent emission of blue light from aequorin (Knight *et al.*, 1996). Treatment of BY-2 cells with sorbitol (400mM) resulted in a rapid transient increase in aequorin luminescence (Fig. 4A) reflecting an increase in [Ca^2+^]_cyt_ of 0.145±0.035 μM (*n*=23). This increase could be inhibited by the presence of Ca^2+^ channel blockers, LaCl_3_ and GdCl_3_, when Ca^2+^ internal store inhibitors (U73122 and dantrolene, Meimoun *et al.*, 2009) showed no significant inhibitory effects (Fig. 4B), indicating that the sorbitol-induced increase in [Ca^2+^]_cyt_ is mainly due to influx of Ca^2+^ across the plasma membrane through Ca^2+^ channels. As the generation of ^1^O_2_ by sorbitol occurred immediately upon hyperosmotic stress (Fig. 2A), the effect of ROS pharmacology on the sorbitol-induced increase in [Ca^2+^]_cyt_ was further tested. The ^1^O_2_ scavenger DABCO strongly reduced the [Ca^2+^]_cyt_ increase compared with that in control cells; the POX inhibitor SHAM showed lower efficiency, while no differences were seen with the O_2_·^–^ scavenger Tiron (Fig. 4A, C). These results strongly suggested that sorbitol-induced Ca^2+^ uptake through Ca^2+^ channels occurs downstream of the sorbitol-induced ^1^O_2_ generation.

In the same way, treatment of BY-2 cells with NaCl (200mM) resulted in a transient increase in aequorin luminescence (Fig. 4D), reflecting an increase in [Ca^2+^]_cyt_ of 0.217±0.059 μM (*n*=23). As for sorbitol, inhibitors of Ca^2+^ release from intracellular organelles (U73122 and dantrolene) failed to suppress the NaCl-induced increase in [Ca^2+^]_cyt_ whereas Ca^2+^ channel blockers LaCl_3_ and GdCl_3_ were efficient at reducing the NaCl-induced increase in [Ca^2+^]_cyt_ (Fig. 4E). In a similar manner to what was observed for sorbitol, this increase was shown to be inhibited by the presence of DABCO (Fig. 4F) but less efficiently by SHAM, whereas Tiron seemed even to increase this Ca^2+^ influx (Fig. 4D, F). The effect of the Ca^2+^ channel inhibitor La^3+^ on NaCl- and sorbitol-induced cell death in BY-2 suspension-cultured cells was then studied. Lanthanum (500 μM) failed to decrease sorbitol- (400mM) and NaCl- (200mM) induced cell death and even increases this cell death after 2h of treatment (Fig. 4G), as observed for DABCO with NaCl (Fig. 3B). These data are in agreement with the link observed between the immediate ^1^O_2_ generation inducing an influx of Ca^2+^ upon sorbitol or NaCl stress and further suggest that these early induced events are not involved in a pathway leading to PCD.

### Hyperosmotic constraints induce change in membrane potential and ion channel activities

**Fig. 5.**
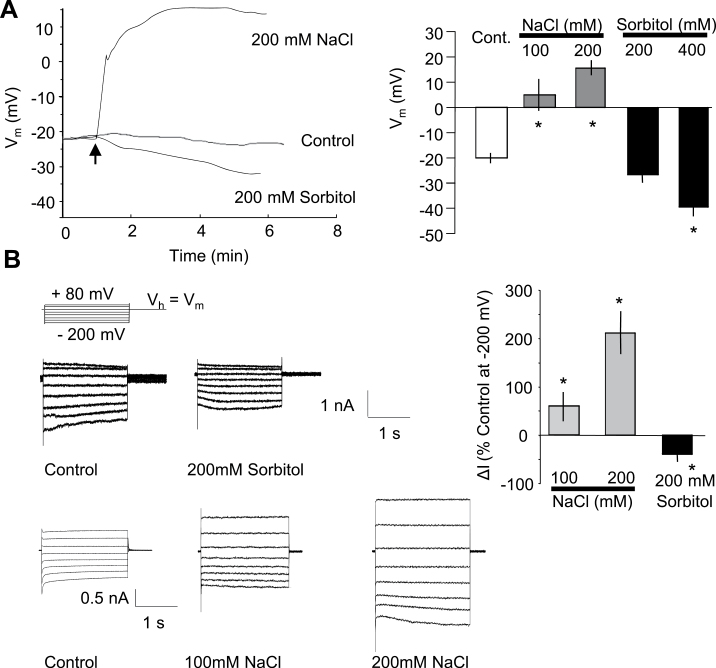
(A) Typical modulation of BY-2 cultured cell plasma membrane (PM) potential variations observed in response to NaCl or sorbitol. (B) Mean values of PM potentials recorded a few minutes after treatment with NaCl (100mM or 200mM) or sorbitol (200mM or 400mM). (C) Typical changes in whole-cell current profiles after treatments with sorbitol (up) or NaCl (down). The protocol was as illustrated; the holding potential (V_h_) was V_m_. (D) Mean values of whole-cell current variations (recorded at –200 mV and 1.8 s) after treatment with NaCl or sorbitol. Current variations are given as a percentage of the control level before treatments. The data correspond to means of at least five independent replicates, and error bars correspond to the SD. *Significantly different from controls, *P* < 0.05.

Saline and non-saline hyperosmotic stresses are well known to modify the plasma membrane potential (*V*_m_) of cells (Teodoro *et al.*, 1998; Zingarelli *et al.*, 1999; Shabala and Cuin, 2008; Wegner *et al.*, 2011). By using an electrophysiological technique (dSEVC), the impact of NaCl and sorbitol on BY-2 cultured cell membrane potential was investigated. In control conditions in culture medium, the *V*_m_ of BY-2 cells was –21.1±2.2 mV (*n*=15). In MS medium, the main ions are 16mM K^+^ and 28mM NO_3_^–^; thus the equilibrium potential estimated for K^+^, *E*_K_, is about –46 mV ([K^+^]_out_=16mM with [K^+^]_in_ estimated at 100mM). The equilibrium potential estimated for NO_3_^–^ is about –25 mV ([NO_3_^–^]_out_=28mM with [NO_3_^–^]_in_ estimated at 5mM). As previously observed with cultured cells of *Arabidopsis thaliana* (Kadono *et al.*, 2010; Tran *et al.*, 2013) or tobacco (Gauthier *et al.*, 2007), the occurrence of anion currents in most of the BY-2 cells in their culture medium could explain the mean polarization of around –20 mV recorded in control and non-stressed conditions. The mean control value of these currents at –200 mV and after 1.8 s of voltage pulse was –1.12±0.2 nA (*n*=11). These currents were shown to be sensitive to structurally unrelated anion channel inhibitors, 9-anthracene carboxylic acid (9-AC) and glibenclamide (gli) (Supplementary Fig. S1 available at *JXB* online), reinforcing the hypothesis of an anionic nature for these currents. Addition of NaCl to suspension cultures resulted in a significant membrane depolarization (Fig. 5A) when sorbitol induced a hyperpolarization of the cells (Fig. 5A), clearly indicating a difference between saline and non-saline hyperosmotic stress. The sorbitol-induced hyperpolarization was correlated with a decrease in anion current (Fig. 5B) when the NaCl-induced depolarization was correlated with a large increase in whole-cell ion current (Fig. 5B). The positive shifts of the reversal potential of the current upon addition of NaCl are in accordance with a current carried by Na^+^.

**Fig. 6.**
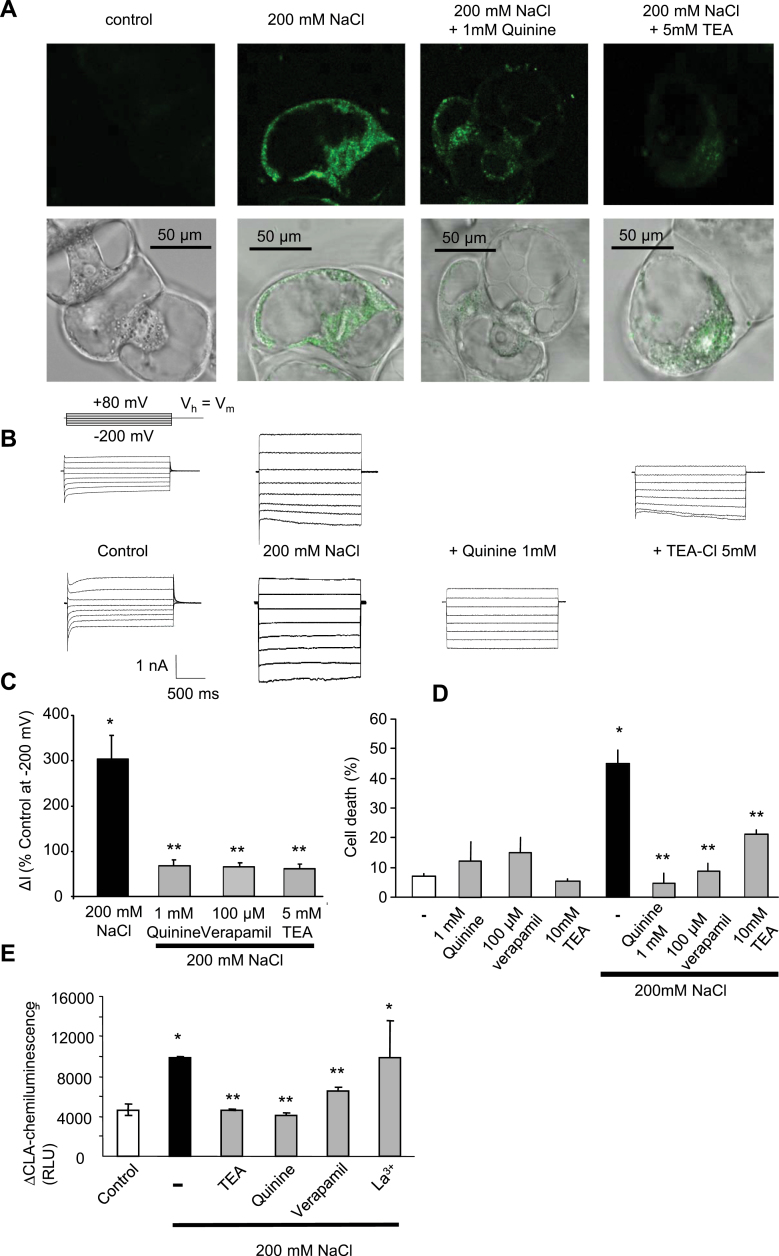
(A) Confocal imaging of NaCl accumulation in BY-2 cells after 1h treatment with 200mM NaCl using the Sodium Green fluorescent probe (left). Decrease in fluorescence in cells pre-treated with quinine (1mM) or TEA^+^ (5mM), blockers of non-selective cation channels (NSCCs), prior to NaCl treatment (right). Corresponding bright field images are shown on the line below. Each image is representative of symptoms observed in at least three independent experiments. (B) Variations of whole-cell currents recorded before and after addition of 200mM NaCl and subsequent addition of 1mM quinine or 5mM TEA^+^. The protocol was as illustrated; the holding potential (V_h_) was V_m_. (C) Mean values of whole-cell current variations (recorded at –200 mV and 1.8 s) after treatment with 200mM NaCl with or without the NSCC blockers quinine (1mM), TEA^+^ (5mM), or verapamil (200 μM). Current variations are given as a percentage of the control level before treatments. The data correspond to means of at least five independent replicates, and error bars correspond to the SD. (D) Effect of pre-treatments with the NSCC blockers on NaCl-induced cell death. The data correspond to means of at least four independent replicates, and error bars correspond to the SD. (E) Effect of pre-treatments with the NSCC blockers or with La^3+^ (500 μM) on NaCl-induced delayed ROS generation and cell death. The data correspond to means of at least five independent replicates, and error bars correspond to the SD. *Significantly different from controls, *P* < 0.05; **significantly different from the NaCl- or sorbitol-treated cells, *P* < 0.05. (This figure is available in colour at *JXB* online.)

The influx of Na^+^ through the plasma membrane by NSCCs was the most probable reason for the cell depolarization (Demidchick *et al.*, 2002*a*, *b*), this Na^+^ uptake was further checked using the Na^+^-sensitive fluorescent probe Sodium Green (Wegner *et al.*, 2011). An accumulation of fluorescence in the cytoplasm of the cells could be observed after 1h treatment with 200mM NaCl (Fig. 6A). This fluorescence could be reduced by using pre-treatments with inhibitors of NSCC, 5mM tetraethylammonium chloride (TEA^+^) or 1mM quinine (Demidchick *et al*., 2002*a*) (Fig. 6A). Addition of 1mM quinine or 5mM TEA^+^ also allowed reduction of the NaCl-induced increase in currents (Fig. 6B, C) in BY-2 cells, as did verapamil (100 μM), another potent inhibitor of NSCCs (Demidchick *et al*., 2002*a*). The impact of these NSCC blockers on the extent of NaCl-induced cell death was thus further tested. The NSCC blockers were efficient at reducing the cell death induced by 200mM NaCl (Fig. 6D), suggesting that NSCC activation is related to the NaCl-induced cell death. As this cell death was also dependent on a delayed O_2_·^–^ generation by NADPH-oxidase (Fig. 3), the effect of the earliest activation of NSCCs by NaCl on this delayed ROS production was also checked. As for cell death, the NSCC blockers were efficient at reducing this NaCl-dependent delayed ROS production (Fig. 6E), suggesting that NaCl influx participates in the O_2_·^–^ generation. Interestingly, the calcium channel blocker La^3+^ did not allow the delayed ROS generation to be decreased, confirming the hypothesis of the induction of different pathways in response to NaCl stress.

### Mitochondrial depolarization is involved in NaCl- but not sorbitol-induced cell death

**Fig. 7.**
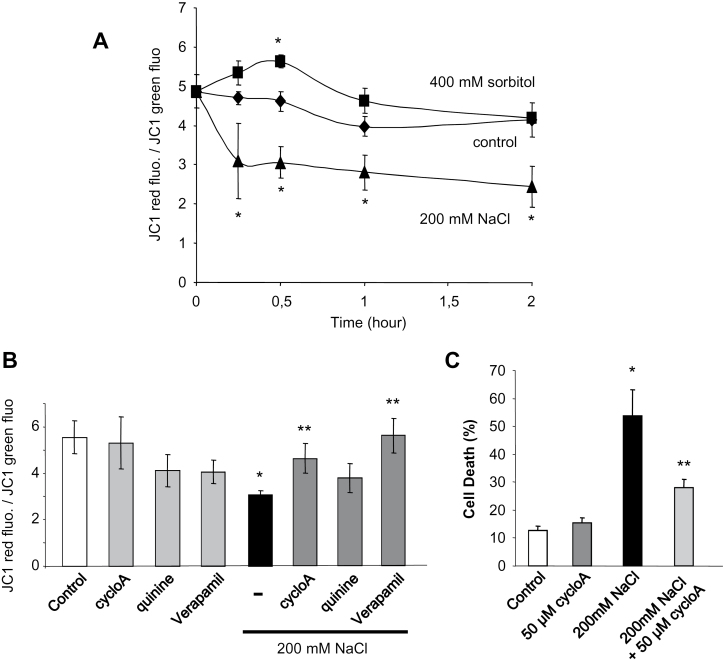
(A) Variations of mitochondrial membrane potential (ΔΨ_m_) of BY-2 tobacco cells after treatments with 200mM NaCl or 400mM sorbitol. (B) Effect of 50 μM cyclosporin A (CsA), an inhibitor of the permeability transition pore, 1mM quinine, or 200 μM verapamil, inhibitors of NSCCs, on ΔΨ_m_ variation induced by 200mM NaCl after 15min. The data reflect the means±SE of at least four independent experiments. (C) Effect of 50 μM CsA, 1mM quinine, or 200 μM verapamil on cell death induced by 200mM NaCl. The data reflect the means±SE of at least three independent replicates. *Significantly different from controls, *P* < 0.05; **significantly different from the NaCl-treated cells, *P* < 0.05.

The role of mitochondria is well recognized in salinity tolerance (Jacoby *et al.*, 2011), and dysfunction of mitochondria, leading to cytochrome *c* release upon salt stress, was shown to induce PCD in *Thellungiella halophila* suspension-cultured cells (Wang *et al.*, 2010). Mitochondria are effectively pivotal in controlling cell life and PCD, through complex mechanisms that culminate in opening of PTPs leading to mitochondrial membrane potential (ΔΨ_m_) loss (Vianello *et al.*, 2007). It was thus checked whether NaCl and sorbitol lead to a decrease in ΔΨ_m_ in the present model. In untreated cells, the JC-1 fluorescence ratio of mitochondria displaying a high ΔΨ_m_ versus mitochondria presenting a low ΔΨ_m_ was greatly superior to 1 (Fig. 7A). This ratio decreased in a time-dependent manner upon addition of NaCl (200mM), indicating that NaCl induced a significant decrease of ΔΨ_m_ in some mitochondria when sorbitol (400mM) induced an increase of ΔΨ_m_ during the first 30min, the ratio reaching the control value after 2h (Fig. 7A). Since a mitochondrial ΔΨ_m_ decrease during cell death was reported to be due to the formation of the mitochondrial PTPs (Vianello *et al.*, 2007), the effect of cyclosporin A (CsA), an inhibitor of PTPs, was tested on NaCl-induced mitochondrial depolarization. A pre-treatment with CsA significantly reduced the NaCl-induced mitochondrial depolarization after 15min (Fig. 7B), indicating that mitochondrial PTPs were involved in NaCl-induced mitochondrial depolarization. Moreover, pre-treatment with CsA significantly inhibited NaCl-induced cell death (Fig. 7C), indicating that PTP formation could participate in NaCl-induced cell death. As the activation of NSCCs occurs rapidly upon NaCl stress (Fig. 5A) and is involved in ROS generation (Fig. 6E), the impact of sodium influx on ΔΨ_m_ was investigated by using NSCC inhibitors. A significant reduction of NaCl-induced mitochondrial depolarization was observed after pre-treatment with verapamil (Fig. 7B). These data show that (i) two different pathways could be involved in the hyperosmotic stress-induced cell death; and (ii) the ROS generation dependent on Na^+^ influx could participate in the decrease in ΔΨ_m_ during NaCl-induced cell death.

## Discussion

As expected from previous studies (Huh, 2002; Wang, 2010), the death of a part of the BY-2 cell population characterized by large cell shrinkage, a hallmark of the PCD process (van Doorn, 2011), was observed in response to NaCl-induced hyperosmotic stress. The extent of cell death was time and dose dependent, reaching about half of the population in 4h with 200mM NaCl. In order to check whether this cell death was due to an active mechanism requiring active gene expression and cellular metabolism, BY-2 cell suspensions were treated with AD, an inhibitor of RNA synthesis, or with Chx, an inhibitor of protein synthesis, prior to NaCl exposure. AD and Chx significantly reduced the NaCl-induced cell death. These results indicated that this cell death required active cell metabolism, namely gene transcription and *de novo* protein synthesis. The same behavior, namely time- and dose-dependent cell death characterized by cell shrinkage and requiring active metabolism using iso-osmotic concentrations of sorbitol, was observed here. This suggests that non-ionic hyperosmotic stress, like ionic hyperosmotic stress, could induce PCD in BY-2 cells as previously observed in various animal cell lines (Murata *et al.*, 2002; Galvez *et al.*, 2003; Niswander and Dokas, 2007). It was then checked whether some early events classically detected during hyperosmotic stress responses and PCD in plant could be involved in this cell death, namely ROS production, an increase in cytosolic Ca^2+^, ion flux variations, and mitochondrial membrane depolarization.

The earliest response observed after exposure of BY-2 cells to NaCl was an immediate peak of ROS. Addition of Tiron (a scavenger of O_2_·^–^) did not significantly reduce NaCl-induced ROS generation, whereas DABCO (a scavenger of ^1^O_2_) avoided this production. Although ^1^O_2_ formation is likely to occur during the exposure to high light intensities (Krieger-Liszkay, 2004), it was also found that different enzymes including POXs could produce extracellular ^1^O_2_ in animals (Kanofsky, 2000; Stief, 2003; Tarr and Valenzeno, 2003) and in plant cells (Kawano *et al.*, 1998; Guo *et al.*, 2009). As the studied cultured cells were non-photosynthetic, the POX inhibitor SHAM was further tested (Kawano *et al.*, 1998; Hossain *et al.*, 2013), and this could reduce the early NaCl-induced ROS generation effectively. Thus, it would appear that these early transient ^1^O_2_ generations are not specific to ionic or non-ionic hyperosmotic stress.

In the case of both NaCl and sorbitol treatments, rapid transient [Ca^2+^]_cyt_ increases in BY-2 cells were observed, as previously described in various models (Knight *et al.*, 1997; Donaldson *et al.*, 2004; Lin *et al.*, 2006; Parre *et al.*, 2007; Ranf *et al.*, 2008). In BY-2 cells, these [Ca^2+^]_cyt_ increases were inhibited by the calcium channel blockers La^3+^ and Gd^3+^ but not by dantrolene and U73122, inhibitors of calcium-induced calcium release channels and of the inositol triphosphate receptor, respectively, known to be efficient in plants (Meimoun *et al.*, 2009). This suggests that the [Ca^2+^]_cyt_ increase was not due to Ca^2+^ release from intracellular Ca^2+^ stores, but to an influx through plasma membrane-permeable Ca^2+^ channels. Although rapid, the Ca^2+^ influxes happened after the ^1^O_2_ generation since DABCO pre-treatments strongly decreased these influxes, suggesting that they were dependent on the generation of ^1^O_2_. Accordingly, SHAM decreased the Ca^2+^ influxes to a lower extent and Tiron was unable to decrease them. This confirms the hypothesis that an ^1^O_2_-dependent Ca^2+^ influx was induced by sorbitol and NaCl and highlights the role of ^1^O_2_ as a signalling molecule (Fischer *et al.*, 2013).

Perturbation of Ca^2+^ homeostasis in plant cells, as well as in animal cells, has been described as a prerequisite for PCD (Grant *et al.*, 2000; Davis and Distelhorst, 2006; Lecourieux *et al.*, 2006). It was not possible to ascertain that in the BY-2 population all cells respond with a Ca^2+^ increase in the face of hyperosmotic stress. However, if only non-dying cells respond with a Ca^2+^ increase, this Ca^2+^ increase has no role in inducing PCD. In other words, if only dying cells (or even all cells) respond with a Ca^2+^ increase, since La^3+^ was inefficient in decreasing NaCl- and sorbitol-induced cell death, and even enhanced this cell death, it could suggest that Ca^2+^ influx could participate in cell protection. After perception of salt stress, the Ca^2+^ spike generated in the cytoplasm of root cells is known to activate the *S*alt *O*verly *S*ensitive (SOS) signal transduction cascade to protect the cells from damage due to excessive ion accumulation (Ji *et al.*, 2013). *SOS3* encodes a myristoylated calcium-binding protein that appears to function as a primary calcium sensor to perceive the increase in cytosolic Ca^2+^ triggered by Na^+^ excess that has entered the cytoplasm. Upon binding to Ca^2+^, SOS3 is able to interact with and activate the protein kinase SOS2 which phosphorylates SOS3 proteins. SOS3–SOS2 interactions recruit SOS2 to the plasma membrane, leading to activation of the downstream target SOS1, an Na^+^/H^+^ antiporter allowing extrusion of excessive Na^+^ from the cytosol (Ji *et al.*, 2013). However, in the present model, neither of these early linked events, Ca^2+^ increase and ^1^O_2_, seemed to be involved in PCD.

On the other hand, Tiron, a scavenger of O_2_·^–^, and DPI, an inhibitor of NADPH-oxidase, decreased the NaCl- and sorbitol-induced cell death and the delayed generation of ROS. This indicated that the delayed and more sustained O_2_·^–^ generation from NADPH-oxidase activity could play a central role in the death of these cells. Several reports implicate NADPH-oxidase activity in production of ROS in salinity stress, with the ROS resulting in Ca^2+^ influx. In the present model, no effect of DPI on early stress-induced Ca^2+^ increase could be detected (Supplementary Fig. S2 available at *JXB* online), in accordance with the delayed DPI-sensitive ROS generation. However, from the present data, a delayed increase in Ca^2+^ linked to NADPH-oxidase activity cannot be excluded, but this increase in Ca^2+^ should be La^3+^ independent, since La^3+^ could not decrease cell death. It is obvious that sorbitol-induced ROS generation could not depend on Na^+^ influx and thus possibly other hyperosmotic-induced events (e.g. NO production) could participate in NADPH-oxidase-dependent ROS generation observed in response to sorbitol and NaCl. However, upon NaCl stress, the delayed ROS generation could be decreased by quinine and verapamil, putative inhibitors of NSCCs (Demidchik *et al.*, 2002*a*, *b*). Even though no definitive molecular candidates have clearly emerged for NSCCs, it seems that various classes of NSCCs could be responsible for influx of Na^+^ under salt stress, especially a depolarization-activated class of NSCCs (Demidchik and Maathuis, 2007). A rapid and large depolarization of the BY-2 cells could be recorded upon NaCl addition due to an increase in a current sensitive to quinine and verapamil but also to TEA^+^, an inhibitor of K^+^ channels known to block some NSCCs (Demidchik *et al.*, 2002*a*). It could also be verified that the accumulation of Na^+^ in the cell was decreased upon pre-treatment with quinine or TEA^+^, strongly suggesting that NSCCs were responsible for Na^+^ influx into BY-2 cells. Moreover, these NSCC blockers were efficient in decreasing the NaCl-induced cell death, highlighting the toxic effect of Na^+^ as previously reported (Huh *et al.*, 2002; Affenzeller *et al.*, 2009; Wang *et al.*, 2010). It is further noticeable that La^3+^, a potent inhibitor of some NSCCs (Demidchick *et al*., 2002*a*, *b*), was unable to decrease NaCl-induced cell death as well as the delayed ROS generation, whereas the NSCC blockers quinine, verapamil, and TEA^+^ failed to decrease the NaCl-induced Ca^2+^ increase (Supplementary Fig. S3 available at JXB online). This indicates that the Ca^2+^ influx was completely dissociated from Na^+^ influx through NSCCs. Moreover, sorbitol- and NaCl-induced Ca^2+^ influxes presented the same characteristics and were induced upon hyperpolarization in the case of sorbitol and depolarization in the case of NaCl, suggesting the voltage independence of the transporter involved. Further studies will be needed to determine if putative ligand-activated calcium channels such as cyclic nucleotide-gated channels or glutamate receptors could be responsible for these Ca^2+^ influxes in BY-2 cells; however, a putative candidate could be the calcium regulatory protein annexin (Laohavisit *et al.*, 2013).

Concerning ion flux regulation in response to sorbitol, a hyperpolarization of the BY-2 cells due to the decrease of anion currents was observed, as previously described in different models (Pennarun and Maillot, 1988; Teodoro *et al.*, 1998; Shabala *et al.*, 2000). Although such a regulation of anion current was shown to be involved during PCD induced by HrpN_ea_, a hypersensitive elicitor from *Erwinia amylovora* (Reboutier *et al.* 2005), it did not seem to be involved in sorbitol-induced PCD since bromotetramisole, an activator of anion channels, failed to limit sorbitol-induced PCD (data not shown), in contrast to the previously observed effect of HrpN_ea_ (Reboutier *et al.*, 2005).

Although ROS-activated outward-rectifying K^+^ channels (KORCs) were shown to be involved in salt-induced PCD (Demidchik *et al*., 2003, 2010), it was not possible to detect rapid activation of KORCs in response to NaCl or sorbitol. However, an activation of KORCs after addition of NaCl cannot be excluded, this current being masked in the large Na^+^ current recorded. On the other hand, KORC activation could also be delayed, as was recently reported in response to O_3_ (Tran *et al.*, 2013).

Finally, one of the most important differences observed between sorbitol- and NaCl-induced PCD in BY-2 cells was the role of mitochondria. Sorbitol did not affect mitochondrial polarization and even slightly and transiently stimulated its polarization, whereas NaCl induced a large mitochondrial depolarization. This depolarization was probably involved in the formation of the PTP since CsA, an inhibitor of PTP, could reduce the NaCl-induced decrease in ΔΨ_m_ as previously described (Wang *et al.*, 2010) and also the NaCl-induced cell death. This could be related to observation made on *Micrasterias denticulate* (Affenzeller *et al.*, 2009) for which a typical laddering of the DNA was only observed for NaCl-stressed cells but not for sorbitol-treated cells. The endonuclease responsible for this laddering could be released from mitochondria through PTPs (Vianello *et al.*, 2007). It is to be noted that the inhibitors of NSCCs, verapamil, quinine, and TEA^+^, were also efficient in limiting the loss in ΔΨ_m_, suggesting a direct role for cytosolic Na^+^-induced mitochondrial dysfunction.

**Fig. 8.**
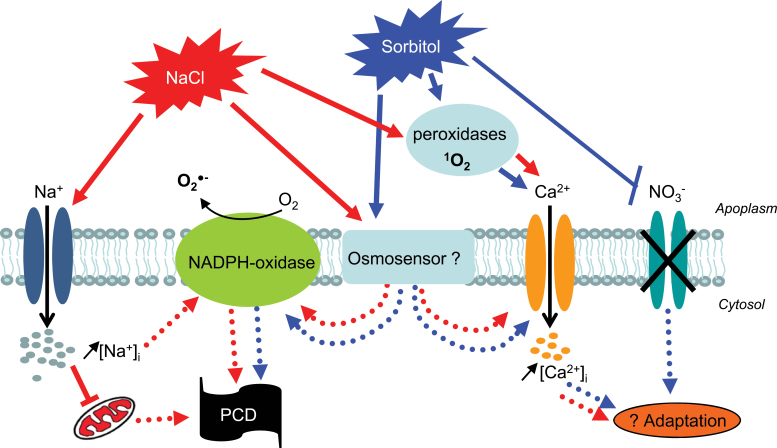
Possible pathways induced by NaCl and sorbitol leading to cell death of tobacco cells. (This figure is available in colour at *JXB* online.)

As already described in numerous models, in BY-2 cells overlapping responses exist in response to ionic and non-ionic hyperosmotic stress and, among them, a PCD process. However, some overlapping responses such as the early transient ^1^O_2_ generation responsible for an influx of Ca^2+^ did not seem to be involved in PCD progress, while other shared responses such as the delayed NADPH-oxidase stimulation were important for these processes (Fig. 8). Specific responses also seemed to be involved in the PCD processes since upon NaCl stress, NADPH-oxidase stimulation was at least partly due to Na^+^ influx through NSCCs, which also seemed to be responsible for mitochondrial depolarization not observed after the sorbitol challenge. Further studies will be needed to characterize fully the pathways leading to PCD induced by these hyperosmotic stresses.

## Supplementary data

Supplementary data are available at *JXB* online.

Fgure S1. Typical anion current recorded in control conditions in BY-2 cells.

Figure S2. NaCl- and sorbitol-induced [Ca^2+^]_cyt_ increase in aequorin-expressing-tobacco BY-2 cells after a pre-treatment with the inhibitor of NADPH-oxidase DPI (20 μM).

Figure S3. NaCl-induced [Ca^2+^]_cyt_ increases in aequorin expressing-tobacco BY-2 cells after pre-treatments with the NSCC blockers quinine, verapamil, or TEA^+^.

Supplementary Data
