# Supplementary material for: Deciphering early events involved in hyperosmotic stress-induced programmed cell death in tobacco BY-2 cells
Source: J Exp Bot. 2014 Jan 13;65(5):1361–75. doi: 10.1093/jxb/ert460 (PMC3969528; doi:10.1093/jxb/ert460)
Supplement: Supplementary Data [file supp_65_5_1361__index.html]

Deciphering early events involved in hyperosmotic stress-induced programmed cell death in tobacco BY-2 cells — Deciphering early events involved in hyperosmotic stress-induced programmed cell death in tobacco BY-2 cells — Supplementary Data 

# Deciphering early events involved in hyperosmotic stress-induced programmed cell death in tobacco BY-2 cells

## Supplementary Data

Data files

**Files in this Data Supplement:**

- Supplementary Data - Supplementary Data
