# Supplementary material for: Deciphering early events involved in hyperosmotic stress-induced programmed cell death in tobacco BY-2 cells
Source: J Exp Bot. 2014 Jan 13;65(5):1361–75. doi: 10.1093/jxb/ert460 (PMC3969528; doi:10.1093/jxb/ert460)
Supplement: Supplementary Data [file supp_ert460_jexbot110460_file001.pdf]

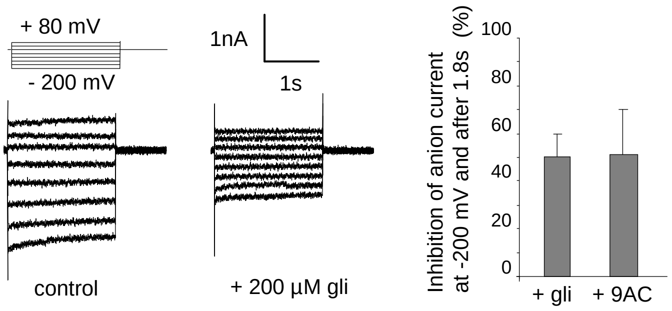

Supplemental figure 1. Typical anion current recorded in control condition in BY-2 cells (left) decreased by anion current inhibitor glibenclamide (center). The protocol was as illustrated, holding potential (Vh) was Vm. Right, mean values of anion current (recorded at -200 mV and 1.8 s) after treatment with anion channel blockers glibenclamide or 9-anthacen carboxilc acid (9AC). Values are given as a percentage of the control level before treatments. The data correspond to means of at least 5 independent replicates and error bars correspond to SD.

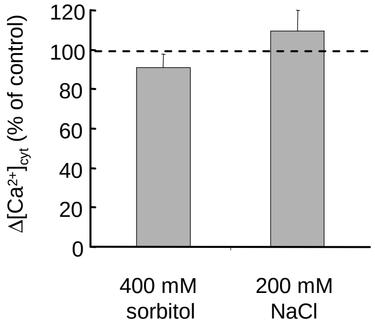

Supplemental figure 3. NaCl- and sorbitol-induced $[Ca_{2+}]_{cyt}$ increase in aequorin expressing-tobacco BY-2 cells after a pretreatment with the inhibitor of the NADPH-oxidase DPI (20 µM). The dashed line refer to the peak level reached after NaCl (200 mM) or sorbitol (400 mM) treatments. Each data point and error bar reflect the mean and SD respectively of at least 3 independent replicates.

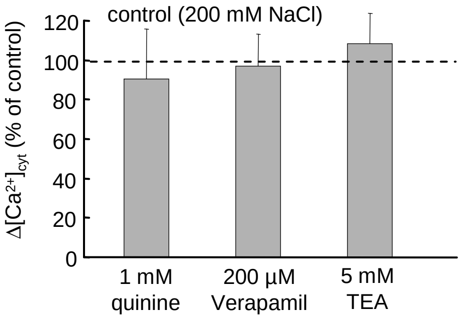

Supplemental figure 2. NaCl-induced $[Ca_{2+}]_{cyt}$ increases in aequorin expressing-tobacco BY-2 cells after pretreatments with the NSCC blockers quinine, verapamil or TEA. The dashed line refer to the peak level reached after NaCl (200 mM) treatment. Each data point and error bar reflect the mean and SD respectively of at least 3 independent replicates.
